# The Temporal Trends of Mortality Due to Tuberculosis in Brazil: Tracing the Coronavirus Disease 2019 (COVID-19) Pandemic’s Effect Through a Bayesian Approach and Unmasking Disparities

**DOI:** 10.3390/microorganisms13051145

**Published:** 2025-05-16

**Authors:** Reginaldo Bazon Vaz Tavares, Dulce Gomes, Thaís Zamboni Berra, Yan Mathias Alves, Antônio Carlos Vieira Ramos, Marcela Antunes Paschoal Popolin, André da Silva Abade, Nathalia Zini, Ariela Fehr Tártaro, Josilene Dália Alves, Fernanda Bruzadelli Paulino da Costa, Maria Eduarda Pagano Pelodan, Beatriz Fornaziero Vigato, Daniele de Moraes Pinheiro, Juliana Queiroz Rocha de Paiva, Clara Ferreira de Souza, Ricardo Alexandre Arcêncio

**Affiliations:** 1 Department of Maternal-Infant and Public Health Nursing, Ribeirão Preto College of Nursing, University of São Paulo, Ribeirão Preto 14040-902, Brazil; reginaldobazon@usp.br (R.B.V.T.); thaiszamboni@live.com (T.Z.B.); yan.alves@usp.br (Y.M.A.); marcela.popolin@mail.uft.edu.br (M.A.P.P.); nathalia_zini@hotmail.com (N.Z.); ariela.ft@usp.br (A.F.T.); fernandabruzadelli@gmail.com (F.B.P.d.C.); mpelodan@usp.br (M.E.P.P.); beatrizfornazierovigato@usp.br (B.F.V.); dani.mpinheiro@usp.br (D.d.M.P.); jqrochapaiva@yahoo.com.br (J.Q.R.d.P.); clarafsouza0211@gmail.com (C.F.d.S.); 2 Department of Mathematics, School of Science and Technology, University of Évora, 7000-671 Évora, Portugal; dmog@uevora.pt; 3 Department of Nursing, State University of Minas Gerais, Passos 96010-440, Brazil; antonio.ramos@uemg.br; 4 Department of Computer Science, Federal Institute of Education, Science and Technology of Mato Grosso, Campus Barra do Garças, Barra do Garças 78600-000, Brazil; andre.abade@ifmt.edu.br; 5 Department of Nursing, Federal University of Mato Grosso, Barra do Garças, Cuiaba 78600-000, Brazil; josilenedalia25@gmail.com

**Keywords:** tuberculosis, mortality, disparities, time series studies, Bayesian analysis, ecological studies

## Abstract

The COVID-19 pandemic disrupted tuberculosis (TB) control, increasing mortality and potentially worsening disparities. This study aimed to analyze the temporal trends of TB mortality in Brazil and to trace the COVID-19 pandemic’s effect using a Bayesian approach, focusing on nationwide data. An ecological study of TB deaths recorded in the Mortality Information System (SIM) from 2012 to 2022 was conducted. Trends and percentage changes in the mortality were estimated. A Bayesian Structural Time Series model combined with an Autoregressive Integrated Moving Average model was used to assess the pandemic’s effect on TB. A total of 51,809 TB deaths were identified, with a mortality rate of 2.27 per 100,000. Higher rates were found among the elderly (6.86), indigenous populations (5.58), and black individuals (4.21). The Bayesian model estimated a 9.9% (CI 8.8–11%) increase in TB mortality due to COVID-19. The Midwest region showed the highest increase (30%, 25–35%). Females experienced a greater post-pandemic monthly increase (2.80%) in mortality than males (0.72%). The Bayesian analysis revealed a significant rise in TB mortality during the COVID-19 pandemic, with notable disparities affecting females, the elderly, the indigenous, and the black populations. These findings highlight the pandemic’s long-term impact on TB and stress the need for equity-focused, data-driven public health responses in Brazil.

## 1. Introduction

Brazil is among the 30 countries with the highest tuberculosis (TB) burden, reporting an incidence rate of 39.7 cases per 100,000 inhabitants in 2024 and a mortality rate of 2.8 deaths per 100,000 in 2023 [1]. Globally, in 2023, an estimated 10.8 million people developed TB, with approximately 1.09 million deaths among HIV-negative individuals and 161,000 among people living with HIV [2].

The COVID-19 pandemic exacerbated this situation by disrupting TB diagnosis and care. The reallocation of health resources, mobility restrictions, fear of infection, and socioeconomic impacts significantly hindered detection and increased loss to follow-up [3,4,5]. These disruptions not only worsened TB outcomes but also led to higher rates of complications and mortality across Brazil.

As a result, TB mortality reached historically high levels, reflecting persistent challenges in healthcare services and control policies, especially among vulnerable populations. Disparities in TB outcomes are evident across age groups, race/skin color, and regions, influenced by socioeconomic and access-related factors. The unequal distribution and quality of healthcare services have likely deepened these disparities in the post-pandemic context [6].

Previous studies have examined the pandemic’s impact on TB in Brazil [4,5,7,8,9], but few have explored its effects on mortality disparities at the national level. To address this gap, analytical approaches such as Bayesian methods can offer a more comprehensive understanding of the pandemic’s influence on TB mortality patterns [10,11].

Health disparities in TB reflect systematic and avoidable differences in mortality across population groups, often linked to social determinants such as income, education, and healthcare access [12,13,14]. Understanding how these disparities evolved during the pandemic is essential for developing targeted policies to reduce TB mortality and improve healthcare equity. This study aimed to analyze the temporal trends of TB mortality in Brazil and to trace the effects of the COVID-19 pandemic using a Bayesian approach.

## 2. Materials and Methods

### 2.1. Design and Setting of This Study

This is an ecological time series study [15] conducted in Brazil.

Brazil is the largest and most populous country in Latin America, with an area of approximately 855,767 square kilometers, divided into five regions: North, Northeast, Midwest, Southeast, and South. It had a population of 203 million people in 2022 [16].

### 2.2. Population, Data Sources, and Selection Criteria

The study population consisted of individuals who died from TB, recorded in the Mortality Information System (SIM) managed by the Department of Informatics of the Unified Health System (DATASUS) in Brazil, during the period from January 2012 to December 2022.

According to the definition provided by the Brazilian Ministry of Health, a death due to TB occurs when TB is the condition that triggers the pathological events leading directly to death [17]. Thus, all deaths in which any form of TB was reported as the underlying cause were considered. Consequently, deaths were selected based on the International Classification of Diseases (ICD-10) codes ranging from A15 to A19.9.

The population data were obtained from the annual estimates of the Brazilian Institute of Geography and Statistics (IBGE) and the 2022 Census, considering the stratification by age, race/skin color, sex, and regions [18].

Regarding age groups, three categories were defined: children and young adolescents (0–14 years), adults (15–59 years), and the elderly (60 years and older) [1]. For race/skin color, the categories adopted by the IBGE were used, namely: yellow (people of Asian descent), white, indigenous, brown (a mix of two or more race/skin color options, excluding yellow), and black [1,19].

### 2.3. Data Analysis

#### 2.3.1. Calculation of the Mortality Rate

The monthly mortality rates for TB were calculated, considering the stratification by age group, sex, race/skin color, and region. For the calculation of mortality rates, the monthly number of deaths due to TB was used as the numerator, and the estimated population for the respective year (2012–2022) served as the denominator, multiplied by the constant 100,000.

Additionally, the annual rates were calculated using the number of TB cases reported in the year as the numerator and the population for that year as the denominator, also multiplied by the constant 100,000.

#### 2.3.2. Trend Estimation

To estimate the trend of the time series, the Seasonal-Trend Decomposition Method using Loess (STL), proposed by Cleveland et al. (1990) [20], was employed. STL is a robust method that uses fitted regression models to decompose a time series into trend, seasonal, and residual components. Assuming an additive decomposition form, the variables of interest (rates) in month t (Yt) are given by the following formula:

Yt = St + Tt + Rt

where St is the seasonal component; Tt is the trend component; and Rt is the residual or noise component.

If a time series is non-stationary, the trend is defined as a long-term growth/decline pattern over a specific period. Seasonality refers to identical patterns that occur at fixed time intervals and with a known frequency, usually caused by seasonal factors. Noise represents the random (or irregular) variations in the series that are not explained by the previous components (regular) [21].

After estimating the three components, only the trend was selected to assess the behavior of the variables of interest over time. The estimation of these components using the STL methodology was conducted using the stl function from the stats package (version 4.6.0, https://stat.ethz.ch/R-manual/R-devel/library/stats/html/stats-package.html (accessed on 10 January 2024)) in the R Cran library (https://cran.r-project.org/web/packages/available_packages_by_name.html (accessed on 10 January 2025)).

#### 2.3.3. Detection of Structural Changes

To analyze whether there were significant changes in the variability of the time series over the period and after the COVID-19 pandemic, the structural change detection technique proposed by Zeileis et al. (2003) [22] was employed.

Basically, a series *y_i_* is considered, and it is assumed that there are m change points in the series, at which the coefficients change from one stable regression relationship to another. Therefore, there are *m* + 1 segments in which the regression coefficients are constant, and the model can be written as follows:

*y_i_* = *x_i_*^⊤^ *βj* + *u_i_*    (*I* = *i*_*j*−1_ + 1, …, *i_j_*,     *j* = 1, …, *m* + 1)

with *x_i_* being the vector of covariates, *βj* (where *j* denotes the segment index) the corresponding regression coefficients, and ui white noise (i.e., an uncorrelated series with a mean of zero and constant variance).

The breakpoints were estimated using the breakpoints function from the strucchange package (version 1.5-4, https://cran.r-project.org/web/packages/strucchange/index.html (accessed on 10 January 2024)) in the R Cran library (https://cran.r-project.org/web/packages/available_packages_by_name.html (accessed on 10 January 2025)). The confidence intervals for the breakpoints were obtained using the confint function, adopting a 95% confidence interval.

#### 2.3.4. Calculation of Percentage Change

To identify the average variations and measure how trends increase or decrease over time, the Average Monthly Percentage Change (AMPC) was calculated for mortality rates by sex, age group, race/skin color, and region. This method allows for the identification of the growth or decline rates of the series.

The calculation was given by the following formula:

$$\mathrm{AMPC}=\frac{\sum_{i=2}^{n}(\frac{Rate\;Month\;i}{Rate\;Month\;i-1}-1)}{n-1}\times 100$$

where *n* denotes the maximum number of months in this study.

For the series that presented significant structural changes, the AMPC calculations were performed considering the dates of the structural changes. This allows for a comparison of the differences in growth or decline rates between the periods in which the data exhibited distinct behaviors.

#### 2.3.5. Calculation of Averages

As an additional way to measure and compare variations between periods and assess the effects of the COVID-19 pandemic, the monthly averages of the TB mortality rates were calculated for all variables. Similar to the calculations of percentage change, the averages were calculated considering the periods indicated by the structural breakpoints in which they were identified.

#### 2.3.6. Assessment of Causal Effect

A Bayesian Structural Time Series (BSTS) model was used in conjunction with Autoregressive Integrated Moving Average (ARIMA) models to infer the causal effect of the COVID-19 pandemic on TB mortality rates in Brazil and its regions.

The BSTS allows for inferring causal impact based on a diffusion–regression state-space model that predicts the counterfactual response of a variable over a synthetic control that would have occurred had no intervention taken place [23]. In this case, the response variable (mortality rate) is a time series, so the causal effect of interest is the difference between the observed series and the series that would have been observed if the intervention had not occurred. To estimate a synthetic control for the comparison period (2020–2022), it is necessary to provide one or more variables that exhibit behavior analogous to that of the studied series in order to obtain the most reliable control possible.

To estimate the counterfactual trends in TB mortality and support the causal inference provided by the BSTS model, we opted to fit ARIMA models to the pre-pandemic period (2010–2019), given the absence of appropriate control time series unaffected by the pandemic. The forecasts generated by the ARIMA models were then used as the counterfactual time series, serving as a synthetic control to estimate the causal impact of COVID-19 on TB mortality during the 2020–2022 period.

The ARIMA models were fitted using the using the Box–Jenkins methodology [24]. This approach allows for the modeling of linear time series based on autoregressive (AR), integrated (I), and moving average (MA) components, defined, respectively, by the parameters p, d, and q. After assessing the stationarity of the series and applying transformations where necessary, we identified the most appropriate ARIMA models based on the Akaike Information Criterion (AIC). Parameter estimation was conducted using maximum likelihood, and residual diagnostics were performed to ensure model adequacy, including checks for autocorrelation, randomness, and normality. The fitted models were then used to forecast expected mortality rates during the pandemic period (2020–2022), serving as synthetic controls in the causal analysis.

We conducted a sensitivity analysis by segmenting the original time series into shorter, consecutive sub-periods and fitting separate ARIMA models to each of these intervals. This procedure allowed us to evaluate whether significant changes occurred in the estimated parameters over time, which could indicate structural instabilities or external influences on the series. Across most sub-periods, the ARIMA parameters remained stable, and when variations were observed, they were minor and did not substantially affect the model’s goodness of fit or predictive accuracy.

Further details on model selection, coefficients, and diagnostics are provided in Supplementary Tables S1 and S2.

The BSTS models were fitted for time series of TB mortality only for the regions of the country, considering a fixed period for the start of the intervention in all regions, defined by the onset of COVID-19 cases in Brazil. The first case of the disease in the country was recorded on 26 February 2020; thus, March 2020 was defined as the start of the intervention for all regions. Consequently, the causal effect analysis was conducted using the same periods.

The BSTS model was implemented using the CausalImpact (version 1.3.0) package [23] (https://cran.r-project.org/web/packages/CausalImpact/index.html (accessed on 10 January 2025)) to estimate the impact of the COVID-19 pandemic on TB mortality. The default model specification was used, which relied on a static intercept and the observed pre-intervention series to generate counterfactual predictions. No explicit trend or seasonal components were included, and no additional covariates were added due to the unavailability of suitable control time series unaffected by the pandemic. The prior standard deviation for the local-level component was set to 0.01, reflecting an assumption of low volatility in the absence of intervention.

The model was fitted using Markov Chain Monte Carlo (MCMC) sampling with 1000 iterations to approximate the posterior distributions of the parameters. Posterior predictive checks were conducted by comparing the observed post-intervention TB mortality data with the model’s counterfactual estimates to assess the model’s fit and validity.

All the analyses were conducted using R Studio version 4.3.2 (PBC, Boston, MA, USA, http://www.rstudio.com/ (accessed on 10 January 2025)).

### 2.4. Ethics Statement

This study was approved by the Research Ethics Committee of the Ribeirão Preto College of Nursing at the University of São Paulo (EERP/USP) under the Certificate of Ethical Appreciation Presentation (CAAE) number 67512823.0.0000.5393.

## 3. Results

Between 2012 and 2022, a total of 51,809 deaths due to tuberculosis (TB) were recorded in Brazil, corresponding to an average mortality rate of 2.27 deaths per 100,000 inhabitants. Table 1 presents the average annual TB mortality rates stratified by sex, age group, race/skin color, and region.

TB mortality was approximately three times higher among males (3.50/100,000) than among females (1.11) and was markedly elevated among older age groups, reaching 6.86 deaths per 100,000 among individuals aged over 59 years. Adults aged 15 to 59 years had an intermediate rate (2.05), while children and adolescents (0–14 years) exhibited very low mortality (0.09).

Marked inequalities were also observed by race/skin color, with indigenous (5.58) and black (4.21) individuals presenting the highest mortality rates, followed by the mixed-race group (2.61). Asian and white populations had the lowest mortality, with rates of 0.73 and 1.49, respectively. At the regional level, the highest average TB mortality rates were observed in the North (2.65), Northeast (2.53), and Southeast (2.36) regions, while the South (1.66) and Midwest (1.50) had the lowest rates during the study period.

### 3.1. Trends and Structural Changes

**Figure 1:**
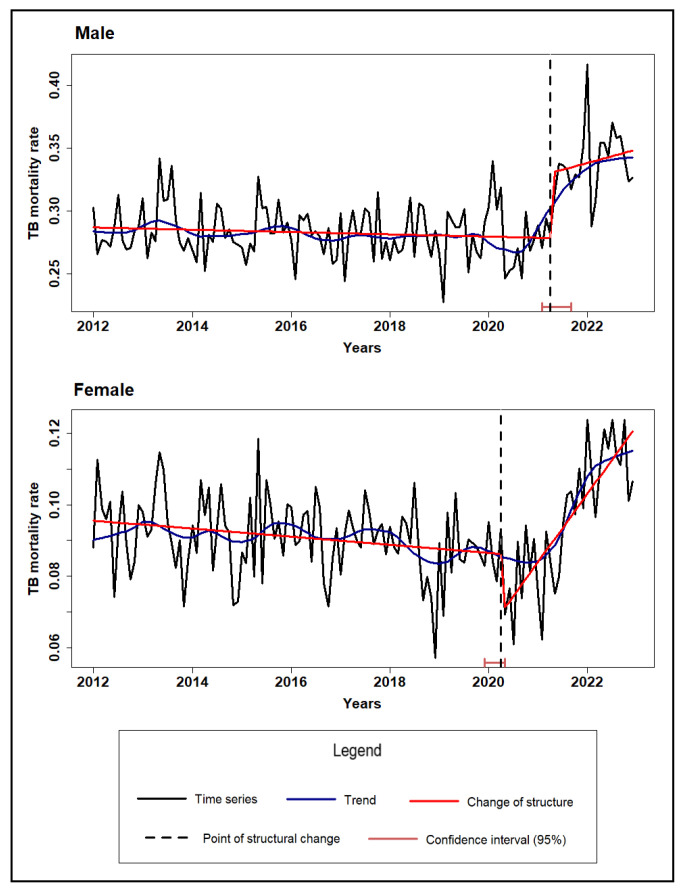
Time series, trend, and structural changes in tuberculosis mortality rate by sex, Brazil (2012–2022).

**Figure 2:**
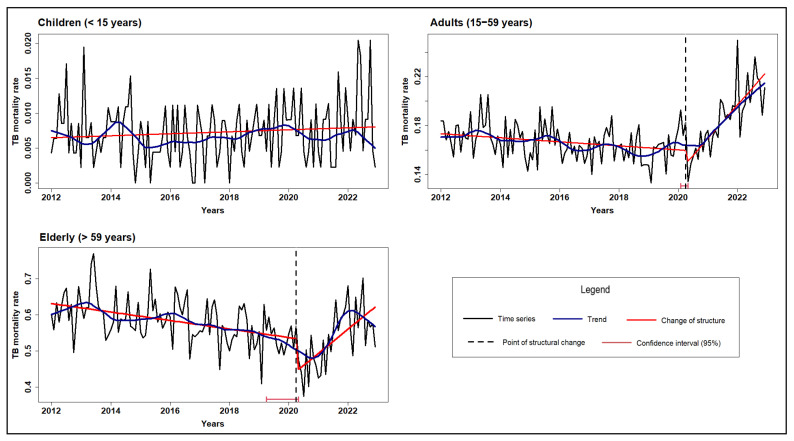
Time series, trend, and structural changes in tuberculosis mortality rate by age group, Brazil (2012–2022).

**Figure 3:**
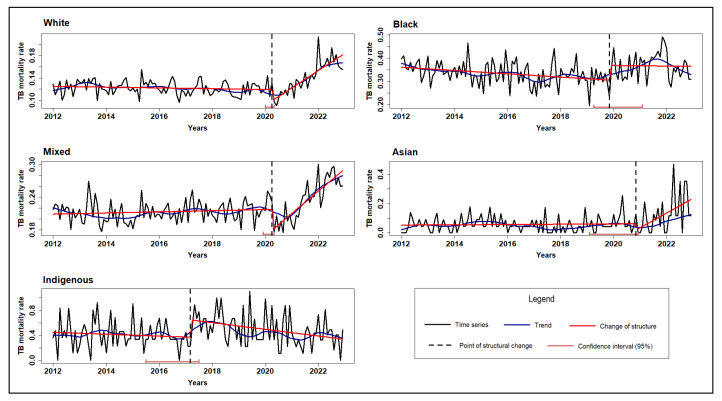
Time series, trend, and structural changes in tuberculosis mortality rate by race/skin color, Brazil (2012–2022).

Figure 1, Figure 2 and Figure 3 illustrate the TB mortality time series, trends, and points of structural change, stratified by sex, age group, and race/skin color.

The trend analyses revealed distinct trends and structural changes across demographic subgroups. In the stratification by sex, male mortality remained consistently higher, but both sexes showed a similar temporal pattern. A gradual decline was observed until early 2020, followed by a reversal and a sharp increase beginning in 2021, suggesting a structural break associated with the COVID-19 pandemic (Figure 1). Regarding the sex variable, the male group showed higher rates throughout the entire period, but both categories exhibited similar behavior, with a reduction in mortality rates in 2020 and a sharp increase starting in 2021 (Figure 1).

The age group analyses showed that TB mortality declined among adults and the elderly up to March 2020, after which structural changes marked by rising mortality were detected. For the youngest age group (0–14 years), mortality remained low and stable, with no evidence of structural change (Figure 2).

Regarding race/skin color, white, mixed-race, and Asian individuals all exhibited structural breaks around the start of the pandemic, followed by an increase in mortality (Figure 3). In contrast, the indigenous population showed a distinct pattern: no structural change was associated with the pandemic; however, a break was observed in 2017, followed by a gradual decline in mortality rates through 2022.

### 3.2. Mean and AMPC

The analysis of mean mortality and AMPC reinforced the patterns found in the trend analysis. Table 2 shows the means and AMPC for the studied variables according to the periods of structural breaks.

Among females, although the absolute mortality rate was lower, the pandemic period was associated with an acceleration in TB mortality, with the AMPC rising from 1.31% before 2020 to 2.80% from May 2020 onward. In males, the AMPC increased more moderately, from 0.41% to 0.72%, but mortality levels remained higher (Table 2).

For adults and the elderly, the AMPC increased substantially in the post-pandemic period, indicating that the reversal of the declining trend was particularly acute in these groups. Among adults aged 15–59 years, the AMPC increased from 0.65% before the pandemic to 2.14% after, while among the elderly it rose from 0.69% to 1.58% (Table 2)

When stratified by race/skin color, white and mixed-race individuals experienced marked increases in TB mortality after the pandemic began, with AMPCs of 2.55% and 2.02%, respectively. The black population showed a persistently increasing trend throughout the study period, with a pre-pandemic AMPC of 1.86% and a post-pandemic value of 1.38%. Although no AMPC could be estimated for the indigenous and Asian populations, the indigenous group maintained the highest average mortality throughout the series, even in the absence of a structural break during the pandemic period.

Geographical disparities were also evident. The North and Northeast regions exhibited the most pronounced structural breaks during the pandemic, with post-2020 AMPCs of 4.64% and 2.63%, respectively—substantially higher than the pre-pandemic trends. The South and Midwest did not exhibit structural breaks associated with COVID-19 but still showed persistent increases in TB mortality, especially in the Midwest, where the AMPC remained high throughout the series (6.33% pre-2019 and 5.39% thereafter). These findings suggest heterogeneous temporal dynamics across regions and demographic groups, reflecting underlying social, structural, and health system-related differences.

### 3.3. Causal Effect Analysis

**Figure 4:**
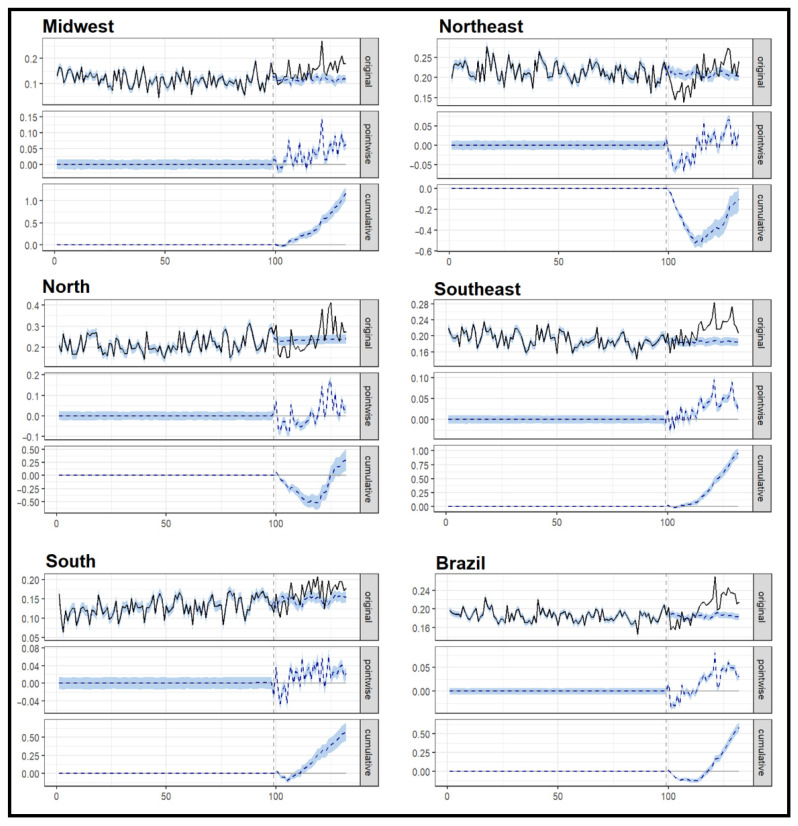
Causal effect analysis on tuberculosis mortality after the COVID-19 pandemic period, by regions, Brazil, 2012–2022. In the first panel (original), the actual values are shown as a solid black line, while the counterfactual values are displayed as a dashed blue line, with 95% posterior probability intervals in light blue. The second panel (pointwise) presents the difference between the observed and predicted values (dashed blue line) along with their 95% posterior probability intervals in light blue. The third panel (cumulative) illustrates the cumulative effect over time following the intervention represented by a dashed blue line with corresponding 95% posterior probability intervals in light blue.

Table 3 and Figure 4 present the results of the BSTS analysis on the causal effects of the COVID-19 pandemic on TB mortality rates by region and at the national level.

At the national level, the BSTS model predicted a mean counterfactual mortality rate of 0.18 deaths per 100,000 inhabitants (95% CI: 0.18–0.19), while the observed mean during the pandemic period was 0.20, indicating an excess mortality of 0.02 per 100,000 and a relative effect of 9.9% (95% CI: 8.8–11%) (Table 3). This suggests that, over the two-year period from (2020–2022), TB mortality in Brazil increased significantly as a consequence of the pandemic.

Regionally, the most substantial effect was observed in the Midwest, where the relative increase reached 30% (95% CI: 25–35%). The Southeast and South regions also experienced notable increases of 16% and 13%, respectively, with predicted mean values significantly below the observed ones. The North region showed a modest but significant increase of 4.1% (95% CI: 1.2–6.8%). In contrast, the Northeast region did not present a consistent increase, with a relative effect of −1.5% (95% CI: −3.2% to 0.06%), suggesting that TB mortality in this region may not have been substantially impacted by the pandemic in the same way as other areas.

The posterior probabilities of a causal effect were high in all regions, ranging from 96.8% in the Northeast to 99.9% in the Midwest, Southeast, and South of Brazil, reinforcing the robustness of the estimated effects.

The graphical representation of the BSTS model results (Figure 4) shows the observed versus predicted mortality trends over time, as well as the pointwise and cumulative differences, which provide further evidence of the interruption in historical trends caused by the pandemic. Taken together, these findings indicate that the COVID-19 pandemic had a heterogeneous impact on TB mortality in Brazil, reversing the previous trends of reduction and exacerbating pre-existing inequalities across demographic and regional lines.

## 4. Discussion

This study aimed to analyze the temporal trends of TB mortality in Brazil and to trace the COVID-19 pandemic’s effect using a Bayesian approach. This study identified 51,809 deaths due to TB in Brazil from 2012 to 2022, corresponding to a mortality rate of 2.14 deaths per 100,000 people in the general population. This study allowed the identification of the groups with the highest mortality rates: elderly individuals (6.86 deaths per 100,000); indigenous populations (5.58 deaths per 100,000), and black individuals (4.21 deaths per 100,000). Furthermore, the Bayesian model estimated a 9.9% increase in TB mortality in Brazil due to the COVID-19 pandemic, and the Midwest region experienced the highest increase in TB mortality, at 30% (CI 95%: 25–35%).

Through the time series analyzed, TB mortality initially declined in 2020 but subsequently increased following the onset of the COVID-19 pandemic. This trend contrasts with findings from a study including 11 countries, in which most countries experienced an early surge in TB mortality in 2020 [25]. The distinct pattern observed in Brazil may be attributable to challenges in accurately capturing TB deaths within the mortality surveillance system during the pandemic. Underreporting and the misclassification of causes of death, exacerbated by the strain on healthcare and reporting infrastructures, may have influenced these findings.

The BSTS analysis showed a cumulative 9.9% (CI 8.8–11%) increase in TB mortality in Brazil over a three-year period compared with a scenario without the COVID-19 pandemic, with significant regional disparities, ranging from a 1.5% (CI −3.2–0.057%) decrease in the Northeast to a 30% (CI 25–35%) increase in the Midwest.

The Midwest saw the highest increase in TB mortality, despite historically low rates and strong control indicators [2,6]. Mortality has risen since 2019, with a time series shift suggesting that the pandemic intensified the pre-existing TB control challenges [5], increasing the disease burden and necessitating further efforts to reverse it.

It is important to note that the Northeast region experienced a significant decline in TB mortality early in 2020, followed by a consistent increase, with a 2.63% change in the mortality rate from June 2020 until the end of 2022. Despite this initial reduction, the region continues to have the highest TB mortality rate in Brazil, suggesting that deaths could continue to rise given the ongoing challenges in TB prevention and treatment. These challenges include insufficiently consolidated municipal TB control programs and limited coverage of the Family Health Strategy [2,26].

The disparities between the regions might also reflect the quality of and access to surveillance systems across the country, influencing the quality of the mortality data. Studies have indicated that, in the North and Northeast regions, mortality system coverage is less than 80% in most municipalities [27], which could lead to underreported TB deaths, especially during the COVID-19 pandemic.

The disproportionate increase in TB mortality among women during the COVID-19 pandemic warrants further attention, as it underscores the intersection of gender and health vulnerability during public health crises. Several factors may have contributed to this trend. First, pandemic-related disruptions, such as the reduced availability of health services, lockdowns, and restrictions on mobility may have disproportionately affected women, who often face structural barriers to accessing care. These barriers were likely intensified by increased caregiving responsibilities during school closures and family illness [28], which limited the time and ability of women to seek timely healthcare.

During the pandemic, these dynamics may have been amplified by a heightened fear of COVID-19 exposure in healthcare settings and competing domestic demands. In many low- and middle-income countries, economic hardship disproportionately affected women working in informal sectors, further limiting their access to transportation or funds for healthcare [28,29].

Additionally, healthcare systems under strain may have prioritized COVID-19 response efforts, reducing the availability of TB diagnostic services. Given the pre-existing underdiagnosis of TB among women [29], these service gaps may have led to further delays or missed diagnoses.

Regarding race/skin color, our result shows that Asian and white individuals had the lowest TB mortality rates in Brazil, although the white population experienced the highest rate increases after the onset of the pandemic. The occurrence of TB among Asian individuals in Brazil is rarely discussed in the literature and represents less than 1% of cases and deaths from the disease in the country [2]. However, the findings indicate a need for further investigation into the occurrence of the disease in this population, considering the increase in deaths after the pandemic.

Black and mixed-race populations in Brazil experience high TB-related mortality rates, highlighting the country’s racial disparities in disease burden [30]. These groups face greater socioeconomic and structural barriers, including inadequate living conditions, lower income levels, and restricted access to healthcare—factors that were further exacerbated by the COVID-19 pandemic [6,31]. Our findings indicate that the TB mortality rate among mixed individuals presented a break with increasing deaths after 2020, reinforcing their status as one of the most affected groups.

The highest mortality rate was among the indigenous population, despite a reduction in mortality since 2017. However, no significant change in the time series was detected as a result of the pandemic. This population has low educational levels, with a high illiteracy rate, and faces substantial barriers to accessing healthcare [32]. Additionally, many live in rural and remote areas, encountering linguistic barriers and prejudice when seeking healthcare services. The difficulty in implementing a multi-cultural approach in healthcare services further contributes to the high mortality in this population [32,33].

The highest TB mortality rate was consistently observed among the indigenous population, despite a gradual reduction since 2017. Notably, no significant structural break was detected in the time series during the COVID-19 pandemic, suggesting that the already elevated mortality in this group remained largely unaffected by the pandemic’s onset. This stability, however, does not indicate resilience but rather points to a persistent pattern of exclusion from health system improvements. The indigenous population in Brazil is characterized by low levels of formal education, with a high rate of illiteracy, and faces considerable barriers to accessing healthcare [32]. These include geographical isolation, as many live in rural and remote areas, as well as cultural and linguistic differences that limit effective communication with health providers. Discrimination and prejudice within the healthcare system also contribute to distrust and delayed care-seeking. Furthermore, the persistent difficulty in implementing culturally sensitive, community-based, and intercultural health approaches continues to undermine the effectiveness of TB control strategies in these communities [32,33]. The absence of a pandemic-related structural break in mortality suggests that the systemic neglect and structural barriers faced by indigenous peoples are long-standing and deeply embedded.

The varying of burden of TB across Brazilian regions is related to the disparities in race/skin color groups’ composition and socioeconomic conditions [6,32]. These factors intersect within specific regional contexts, shaping distinct patterns of vulnerability [6,32]. Race/skin color and income-related disparities in TB highlight the urgency of addressing these inequalities through targeted public health strategies. Aligning these efforts with the Sustainable Development Goals (SDGs) is critical, particularly Goal 10 of reducing inequality. Additionally, Goal 3, which aims to end the TB epidemic by 2030, underscores the need for comprehensive and equitable interventions. Addressing these disparities is vital for reducing TB mortality and advancing health equity [34].

Regarding age groups, no significant structural changes were observed in the time series of TB mortality among children. However, an increase in deaths was noted beginning in 2022, suggesting potential shifts in disease dynamics. This trend aligns with the broader pattern of TB being more prevalent among adults in the economically active age group, both globally and in Brazil [1,2]. Notably, our findings indicate that these adult groups experienced a significant rise in mortality following the pandemic.

Before the pandemic, the elderly presented a declining trend in mortality, but COVID-19 appears to have reversed this pattern, suggesting a possible interaction between the two diseases. Elderly individuals have the highest TB mortality rate, with most cases resulting from the reactivation of old lesions [35]. The reactivation of TB in older adults may have been driven by the impact of COVID-19 on individuals with weakened health. Additionally, the prioritization of COVID-19 in elderly populations may have led to the underreporting of TB cases, resulting in delayed or missed diagnoses and, consequently, increased mortality. This scenario highlights the need for targeted strategies to improve TB detection and management in elderly individuals.

Co-infection between TB and COVID-19 may exacerbate disease severity through immune system compromise and has been linked to delays in diagnosis and treatment, potentially worsening outcomes [36,37]. These interactions are particularly concerning in areas with a high COVID-19 incidence, where overwhelmed health systems may have further disrupted TB care. While this study focused on population-level mortality trends, the lack of clinical data limited a more detailed analysis of the co-infection dynamics. Future studies incorporating case-level clinical and spatial data are essential to explore these effects and inform integrated public health responses.

Although the findings of this study are significant, they should be interpreted with caution due to certain limitations. The results rely on secondary data, which may contain inconsistencies that could affect the results presented. Moreover, the COVID-19 pandemic likely contributed to the underreporting of TB deaths, potentially distorting mortality estimates.

Another consideration is that the time series models did not incorporate covariates, such as socioeconomic conditions or health system factors, which may have influenced the accuracy of the estimated mortality after the pandemic. While this study infers the impact of the pandemic based on temporal variations, it may not fully account for the influence of other concurrent factors. Despite these challenges, the data and the analytical approach provide valuable evidence to support public policy decisions, improve resource allocation, and enhance TB control efforts.

## 5. Conclusions

The COVID-19 pandemic significantly exacerbated TB mortality rates, with disproportionate effects on vulnerable groups, including women, elderly individuals, indigenous populations, and black individuals. These findings emphasize the long-term impact of the pandemic on TB control efforts and highlight the need for targeted interventions to address health disparities. This study provides critical insights for policymakers to develop strategies aimed at mitigating the pandemic’s impact on TB mortality and reducing health inequities in Brazil.

## Figures and Tables

**Table 1:** Average annual mortality rate of tuberculosis by sex, age group, race/skin color, and region, Brazil, 2012–2022.

Variable	Annual Mortality Rate (Deaths/100,000 Inhabitants)
**Sex**	
Male	3.50
Female	1.11
**Age group (Years)**	
0–14	0.09
15–59	2.05
>59	6.86
**Race/skin color**	
White	1.49
Black	4.21
Mixed	2.61
Indigenous	5.58
Asian	0.73
**Region**	
North	2.65
South	1.66
Northeast	2.53
Southeast	2.36
Midwest	1.50

**Table 2:** AMPC and mean of tuberculosis mortality rates for sex, age group, race/skin color, region, Brazil, 2012–2022.

Variable	Period (Month, Year)	AMPC (%) *	Mean
**Sex**			
Male	January 2012–April 2021	0.41	0.2829
	May 2021–December 2022	0.72	0.3383
Female	January 2012–April 2020	1.31	0.0907
	May 2020–December 2022	2.80	0.0941
**Age group (Years)**			
0–14	January 2012–December 2022	-	0.0072
	-	-	-
15–59	January 2012–April 2020	0.65	0.1676
	May 2020–December 2022	2.14	0.1833
>59	January 2012–April 2020	0.69	0.5800
	May 2020–December 2022	1.58	0.5267
**Race/skin color**			
White	January 2012–April 2020	0.90	0.1225
	May 2020–December 2022	2.55	0.1383
Black	January 2012–May 2019	1.86	0.3333
	December 2019–December 2022	1.38	0.3654
Mixed	January 2012–April 2020	0.72	0.2147
	May 2020–December 2022	2.02	0.2312
Indigenous	January 2012–March 2017	-	0.3968
	March 2017–December 2022	-	0.4983
Asian	January 2012–November 2020	-	0.0585
	December 2020–December 2022	-	0.0892
**Region**			
North	January 2012–April 2020	3.00	0.2192
	May 2020–December 2022	4.64	0.2365
South	January 2012–December 2022	2.85	0.1382
	-	-	-
Northeast	January 2012–May 2020	0.89	0.2151
	June 2020–December 2022	2.63	0.2011
Southeast	January 2012–October 2018	0.53	0.1939
	November 2018–December 2022	1.13	0.1962
Midwest	January 2012–February 2019	6.33	0.1106
	March 2019–December 2022	5.39	0.1436

* AMPC—Average Monthly Percentage Change.

**Table 3:** Causal effects on tuberculosis mortality after the COVID-19 pandemic period, by regions, Brazil, 2012–2022.

Variable (*p*-Value)	Average	Cumulative
Brazil (*p* = 0.001)		
Actual	0.2	6.7
Prediction (95% CI *)	0.18 [0.18–0.19]	6.10 [6.04–6.16]
Absolute effect (95% CI)	0.018 [0.016–0.02]	0.601 [0.541–0.67]
Relative effect (95% CI)	9.9% [8.8–11%]	9.9% [8.8–11%]
Midwest (*p* = 0.001)		
Actual	0.15	5.04
Prediction (95% CI)	0.12 [0.11–0.12]	3.88 [3.73–4.02]
Absolute effect (95% CI)	0.035 [0.031–0.04]	1.169 [1.021–1.31]
Relative effect (95% CI)	30% [25–35%]	30% [25–35%]
Northeast (*p* = 0.045)		
Actual	0.21	6.82
Prediction (95% CI)	0.21 [0.21–0.21]	6.92 [6.82–7.05]
Absolute effect (95% CI)	−0.0031 [−0.0069–−0.00012]	−0.1021 [−0.2271–0.00387]
Relative effect (95% CI)	−1.5% [−3.2–0.057%]	−1.5% [−3.2–0.057%]
North (*p* = 0.004)		
Actual	0.24	8.06
Prediction (95% CI)	0.23 [0.23–0.24]	7.74 [7.54–7.96]
Absolute effect (95% CI)	0.0095 [0.003–0.016]	0.3136 [0.098–0.512]
Relative effect (95% CI)	4.1% [1.2–6.8%]	4.1% [1.2–6.8%]
Southeast (*p* = 0.001)		
Actual	0.21	7.07
Prediction (95% CI)	0.18 [0.18–0.19]	6.10 [6.01–6.20]
Absolute effect (95% CI)	0.03 [0.026–0.032]	0.97 [0.874–1.066]
Relative effect (95% CI)	16% [14–18%]	16% [14–18%]
South (*p* = 0.001)		
Actual	0.17	5.47
Prediction (95% CI)	0.15 [0.14–0.15]	4.84 [4.71–4.96]
Absolute effect (95% CI)	0.019 [0.015–0.023]	0.629 [0.506–0.756]
Relative effect (95% CI)	13% [10–16%]	13% [10–16%]

* 95% CI—confidence interval.

## Data Availability

The original contributions presented in this study are included in this article/Supplementary Materials. Further inquiries can be directed to the corresponding author. The raw data supporting the conclusions of this article are available in the Supplementary Materials.

## Supplementary Materials

The following supporting information can be downloaded at https://www.mdpi.com/article/10.3390/microorganisms13051145/s1, The content in the files are the dataset used in the analysis. Table S1: Analysis of residuals of temporal modeling of mortality rates, Brazil (2012–2022); Table S2: Predictive analysis of the mortality rates models, Brazil (2012–2022).

## Abbreviations

The following abbreviations are used in this manuscript:

TB	Tuberculosis
SIM	Mortality Information System
WHO	World Health Organization
DATASUS	Department of Informatics of the Unified Health System
IBGE	Brazilian Institute of Geography and Statistics
STL	Seasonal-Trend Decomposition by Loess
BSTS	Bayesian Structural Time Series
ARIMA	Autoregressive Integrated Moving Average
CAAE	Certificate of Presentation for Ethical Appreciation
SDG	Sustainable Development Goal
CI	Confidence interval
AIC	Akaike Information Criterion
ICD-10	International Classification of Diseases

## References

[1] Ministry of Health (2025). Boletim Epidemiológicol.

[2] World Health Organization Global Tuberculosis Report, 2024. https://iris.who.int/bitstream/handle/10665/363752/9789240061729eng.pdf?sequence=1.

[3] Hino P., Yamamoto T.T., Magnabosco G.T., Bertolozzi M.R., Taminato M. (2021). Impacto da COVID-19 no controle E reorganização da atenção à tuberculose. Acta Paul. Enferm..

[4] Berra T.Z., Ramos A.C.V., Alves Y.M., Tavares R.B.V., Tartaro A.F., do Nascimento M.C., Moura H.S.D., Delpino F.M., de Almeida Soares D., Silva R.V.D.S. (2022). Impact of COVID-19 on Tuberculosis Indicators in Brazil: A Time Series and Spatial Analysis Study. Trop. Med. Infect. Dis..

[5] Blumea M.C., Waldmanb E.A., Lindosoc A.A.B.P., Rújulac M.J.P., Orlandic G.M., de Lourdes Viude Oliveirac M., Guimarãesa A.M.S. (2024). The impact of the SARS-CoV-2 pandemic on tuberculosis notifications and deaths in the state of São Paulo, Brazil: A cross-sectional study. Lancet Reg. Health Am..

[6] Cortez A.O., Melo A.C., Neves L.O., Resende K.A., Camargo P. (2021). Tuberculosis in Brazil: One country, multiple realities. J. Bras. Pneumol..

[7] de Souza C.D.F., Neto E.R.D., Matos T.S., Ferreira A.C.F., Bezerra-Santos M., da Silva Junior A.G., Carmo R.F.D. (2023). Bridging the Gaps: Investigating the Complex Impact of the COVID-19 Pandemic on Tuberculosis Records in Brazil. Trop. Med. Infect. Dis..

[8] Tavares R.B.V., Berra T.Z., Alves Y.M., Popolin M.A.P., Ramos A.C.V., Tártaro A.F., de Souza C.F., Arcêncio R.A. (2024). Unsuccessful tuberculosis treatment outcomes across Brazil’s geographical landscape before and during the COVID-19 pandemic: Are we truly advancing toward the sustainable development/end TB goal?. Infect Dis. Poverty.

[9] Hentringer I.M.B., da Mata Ribeiro J.A., de Jesus Brandão Barreto I., de Santana Cabral Silva A.P. (2022). Effect of COVID-19 Pandemic on New Cases of Tuberculosis in Brazil: A Temporal and Spatial Analysis. Mundo Saude.

[10] Antunes J.L.F., Cardoso M.R.A. (2015). Using time series analysis in epidemiological studies. SciELO.

[11] Martinez E.Z., Achcar J.A. (2014). Trends in epidemiology in the 21st century: Time to adopt bayesian methods. Cad. Saude Publica.

[12] World Health Organization The End TB Strategy, 2015. https://iris.who.int/bitstream/handle/10665/331326/WHO-HTM-TB-2015.19eng.pdf?sequence=1.

[13] Agency for Healthcare Research and Quality (2021). National Healthcare Quality and Disparities Report.

[14] Duarte R., Lönnroth K., Carvalho C., Lima F., Carvalho A.C.C., Muñoz-Torrico M., Centis R. (2018). Tuberculosis, social determinants and co-morbidities (including HIV). Pulmonology.

[15] Morgenstern H. (1995). Ecologic studies in epidemiology: Concepts, principles, and methods. Annu. Rev. Public Health.

[16] Brazilian Institute of Geography and Statistics (IBGE) (2022). Brazil em Síntese.

[17] Ministry of Health (2019). Manual de Recomendações para o Controle da Tuberculose no Brasil Brasília.

[18] Brazilian Institute of Geography and Statistics (IBGE) (2022). População.

[19] Brazilian Institute of Geography and Statistics (IBGE) (2022). Censo Demográfico 2022. Identificação Étnico-Racial Da População, Por Sexo E Idade.

[20] Cleveland R.B., Cleveland W.S. (1990). STL: A Seasonal-Trend Decomposition Procedure Based on Loess. J. Off. Stat..

[21] Brockwell P.J., Davis R.A. (2002). Introduction to Time Series and Forecasting.

[22] Zeileis A., Kleiber C., Krämer W., Hornik K. (2003). Testing and dating of structural changes in practice. Comput. Stat. Data Anal..

[23] Brodersen K.H., Gallusser F., Koehler J., Remy N., Scott S.L. (2015). Inferring causal impact using Bayesian structural time-series models. Ann. Appl. Stat..

[24] Box G.E.P., Jenkins G.M. (1976). Time Series Analysis: Forecasting and Control.

[25] Nalunjogi J., Mucching-Toscano S., Sibomana J.P., Centis R., D'Ambrosio L., Alffenaar J.W., Denholm J., Blanc F.X., Borisov S., Danila E. (2023). Impact of COVID-19 on diagnosis of tuberculosis, multidrug-resistant tuberculosis, and on mortality in 11 countries in Europe, Northern America, and Australia. A Global Tuberculosis Network study. Int. J. Infect Dis..

[26] Brito A.B., Magalhães W.B., de Paiva J.P.S., de Leal T.C., Silva L.F., da Santos L.G., Santana G.B.A., Fernandes T.R.M.O., Souza C.D.F. (2020). Tuberculosis in Northeastern Brasil (2001–2016): Trend, clinical profile, and prevalence of risk factors and associated comorbidities. Rev. Assoc. Med. Bras..

[27] Szwarcwald C.L., Morais Neto O.L., Frias P.G., Souza P.R.B., Cortez-Escalante J.J., Lima R.B., Viola R.C. (2011). Busca ativa de óbitos e nascimentos no Nordeste e na Amazônia Legal: Estimação das coberturas do SIM e do SINASC nos municípios brasileiros. Departamento de Análise de Situação de Saúde, Secretaria de Vigilância em Saúde, Ministério da Saúde, Organizadores. Saúde Brasil, 2010: Uma Análise Da Situação de Saúde E de Evidências Selecionadas de Impacto de ações de Vigilância Em Saúde.

[28] UN Women (2020). The Impact of COVID-19 on Women.

[29] Horton K.C., MacPherson P., Houben R.M.G.J., White R.G., Corbett E.L. (2016). Sex Differences in Tuberculosis Burden and Notifications in Low- and Middle-Income Countries: A Systematic Review and Meta-Analysis. PLoS Med..

[30] de Sousa Viana P.V., Paiva N.S., Villela D.A.M., Bastos L.S., de Souza Bierrenbach A.L. (2020). Paulo Cesar Basta Factors associated with death in patients with tuberculosis in Brazil: Competing risks analysis. PLoS ONE.

[31] Brazil Brazilian Institute of Geography Statistics (I.B.G.E.) (2023). Pessoas Pretas e Pardas Continuam Com Menor Acesso a Emprego Educação Segurança e Saneamento.

[32] Basta P.C., Marques M., de Oliveira R.L., Cunha E.A.T., da Costa Resendes A.P., Souza-Santos R. (2013). Social inequalities and tuberculosis: An analysis by race/color in Mato Grosso do Sul, Brazil. Rev. Saúde Pública.

[33] Vaz I., Paiva N.S., Sousa P.V. (2023). Spatial-temporal evolution of tuberculosis incidence rates in indigenous and non-indigenous people of Brazil, from 2011 to 2022. Rev. Bras. Epidemiol..

[34] Satyanarayana S., Thekkur P., Kumar A.M.V., Lin Y., Dlodlo R.A., Khogali M., Zachariah R., Harries A.D. (2020). An Opportunity to END TB: Using the Sustainable Development Goals for Action on Socio-Economic Determinants of TB in High Burden Countries in WHO South-East Asia and the Western Pacific Regions. Trop. Med. Infect. Dis..

[35] Caraux-Paz P., Diamantis S., de Wazières B., Gallien S. (2021). Tuberculosis in the Elderly. J. Clin. Med..

[36] Di Bari V., Cerva C., Libertone R., Carli S.M., Musso M., Goletti D., Aiello A., Mazzarelli A., Cannas A., Matusali G. (2025). Impact of Severity of COVID-19 in TB Disease Patients: Experience from an Italian Infectious Disease Referral Hospital. Infect. Dis. Rep..

[37] Casco N., Jorge A.L., Palmero D.J., Alffenaar J.W., Fox G.J., Ezz W., Cho J.G., Denholm J., Skrahina A., Solodovnikova V. (2023). Long-term outcomes of the global tuberculosis and COVID-19 co-infection cohort. Eur. Respir. J..

