# Supplementary material for: The Temporal Trends of Mortality Due to Tuberculosis in Brazil: Tracing the Coronavirus Disease 2019 (COVID-19) Pandemic’s Effect Through a Bayesian Approach and Unmasking Disparities
Source: Microorganisms. 2025 May 16;13(5):1145. doi: 10.3390/microorganisms13051145 (PMC12114275; doi:10.3390/microorganisms13051145)
Supplement: Supplementary file 1 [file microorganisms-13-01145-s001.zip › Description of supplementary dataset.pdf]

The Temporal Trends of Mortality Due to Tuberculosis in Brazil: Tracing the Coronavirus Disease 2019 (COVID-19) Pandemic's Effect Through a Bayesian Approach and Unmasking Disparities

Description of supplementary dataset

We provided four datasets as supplementary materials, containing the data used for the study's analysis. The datasets are available as .csv (comma-separated values) files for easier access.

The first dataset (1_temp_age_group) includes the number of deaths and the mortality rate per month, categorized by age group. The second dataset (2_temp_geo_regions) includes the number of deaths and the mortality rate per month, categorized by geographic region. The third dataset (3_temp_race_color) includes the number of deaths and the mortality rate per month, categorized by race/color. The fourth dataset (4_temp_sex) includes the number of deaths and the mortality rate per month, categorized by sex.
