# Supplementary material for: The Temporal Trends of Mortality Due to Tuberculosis in Brazil: Tracing the Coronavirus Disease 2019 (COVID-19) Pandemic’s Effect Through a Bayesian Approach and Unmasking Disparities
Source: Microorganisms. 2025 May 16;13(5):1145. doi: 10.3390/microorganisms13051145 (PMC12114275; doi:10.3390/microorganisms13051145)
Supplement: Supplementary file 1 [file microorganisms-13-01145-s001.zip › Supplementary_tables.pdf]

Temporal trends of mortality due to tuberculosis in Brazil: tracing COVID-19's pandemic effect through a Bayesian approach and unmasking disparities

SUPPLEMENTARY MATERIALS

Table of contents

SUPPLEMENTARY TABLES 3

Supplementary Table S1 .... **Error! Bookmark not defined.**

Supplementary Table S2 - 4

SUPPLEMENTARY TABLES

Supplementary Table S1 - Analysis of residuals of temporal modeling of mortality rates, Brazil (2012–2022)

Region / Tests	Test statistics	P-value
**Midwest**		
Ljung-Box	6.03	0.81
Box-Pierce	5.49	0.86
Turning Point Test	0.97	0.33
Kolmogorov-Smirnov	0.04	0.99
T test for the means	-0.90	0.37
**Northeast**		
Ljung-Box	5.01	0.89
Box-Pierce	4.61	0.91
Turning Point Test	-0.48	0.63
Kolmogorov-Smirnov	0.15	0.88
T test for the means	-0.95	0.35
**North**		
Ljung-Box	1.10	0.99
Box-Pierce	1.02	0.99
Turning Point Test	0.16	0.87
Kolmogorov-Smirnov	0.12	0.15
T test for the means	0.003	0.99
**Southeast**		
Ljung-Box	7.65	0.66
Box-Pierce	7.09	0.72
Turning Point Test	-1.12	0.26
Kolmogorov-Smirnov	0.05	0.96
T test for the means	-1.36	0.18
**South**		
Ljung-Box	6.26	0.79
Box-Pierce	5.80	0.83
Turning Point Test	1.28	0.20
Kolmogorov-Smirnov	0.11	0.19
T test for the means	0.97	0.34
**Brazil**		
Ljung-Box	8.28	0.60
Box-Pierce	7.64	0.66
Turning Point Test	0.32	0.75
Kolmogorov-Smirnov	0.06	0.89
T test for the means	-0.43	0.67

After verifying the significance of the models parameters and considering the lowest Akaike Information Criterion (AIC) values, the models that seem to be the most suitable in terms of their ability to describe data variability over time, as well as their good forecasting performance, were: Midwest: SARIMA(0,1,1)(5,0,2); Northeast: SARIMA(2,1,1)(1,0,2); North: SARIMA(1,0,1)(1,0,0); Southeast: SARIMA(1,1,1)(2,0,0); South: SARIMA(0,1,1)(1,0,2); Brazil: SARIMA(1,0,0)(2,0,0).

The residual analysis of the models (Table S1) shows that all are consistent with the necessary assumptions (independent and

identically distributed, with a normal distribution of zero mean and constant variance).

Supplementary Table S2 - Predictive analysis of the mortality rates models, Brazil (2012–2022)

Test	Midwest	Northeast	North	Southeast	South	Brazil
RMSE	0.02	0.02	0.04	0.02	0.02	0.01
MAE	0.02	0.02	0.03	0.01	0.02	0.01
MAPE	18.24	7.83	15.47	7.07	13.90	4.91

RMSE - Root Mean Squared Error; MAE - Mean Absolute Error; MAPE - Mean Absolute Percentage Error.

The predictive analysis of the models revealed low accuracy measures (RMSE, MAE, and MAPE) (Table S2) and good predictive ability. For the Midwest and North, where the models showed 18.24% and 15.47% error compared to the actual data, they can be considered quite reasonable.
